# Overview of EBV-Associated T/NK-Cell Lymphoproliferative Diseases

**DOI:** 10.3389/fped.2018.00417

**Published:** 2019-01-04

**Authors:** Hiroshi Kimura, Shigeyoshi Fujiwara

**Affiliations:** ^1^Department of Virology, Nagoya University Graduate School of Medicine, Nagoya, Japan; ^2^Department of Allergy and Clinical Immunology, National Research Institute for Child Health and Development, Tokyo, Japan; ^3^Division of Hematology and Rheumatology, Department of Medicine, Nihon University School of Medicine, Tokyo, Japan

**Keywords:** EBV-T/NK-LPDs, chronic active EBV infection, hydroa vacciniforme-like LPD, severe mosquito bite allergy, extranodal NK/T-cell lymphoma, aggressive NK-cell leukemia

## Abstract

Epstein-Barr virus-associated T/natural killer-cell lymphoproliferative diseases (EBV-T/NK-LPDs) are a group of rare diseases resulting from ectopic infection of T or natural killer (NK) lymphocytes with Epstein-Barr virus (EBV). EBV-T/NK-LPDs include chronic active EBV infection, EBV-associated hemophagocytic lymphohistiocytosis, hydroa vacciniforme-like lymphoproliferative disease, and severe mosquito bite allergy. Extra-nodal NK/T-cell lymphoma-nasal type and aggressive NK-cell leukemia can also be included in this broad spectrum. Currently, the etiology of EBV-T/NK-LPDs is unknown and no curative therapy has been established, except for hematopoietic stem cell transplantation. While most cases of EBV-T/NK-LPDs have been documented in specific areas of the world, they have also been documented more broadly across East Asia and Latin America. Consequently, active research and discussion of EBV-T/NK-LPDs are both necessary and important within the extensive international community of scientists and clinicians, to elucidate their etiology and develop a standard therapy.

## Introduction

Epstein-Barr virus (EBV) is a ubiquitous gammaherpesvirus that persistently infects more than 90% of the world's adult population. EBV infection primarily targets B cells and epithelial cells. Although infection with EBV is usually asymptomatic, the development of symptomatic disease has been associated with a delayed primary infection leading to infectious mononucleosis in adolescents and young adults, and with various EBV-associated malignancies. The virus also causes various opportunistic diseases in immunocompromised hosts.

In apparent immunocompetent hosts, EBV can also induce chronic disease with prolonged infectious mononucleosis-like symptoms and a sustained EBV DNA load in the peripheral blood. Although rare, this disease has been called chronic active EBV disease or chronic active EBV infection; both are abbreviated as CAEBV (1, 2). Interestingly, while EBV-infected B-cell proliferation has been observed in most patients with CAEBV in Western countries, the virus is found mainly in T or NK cells proliferating clonally in East Asian countries, including Japan (3, 4).

In addition to CAEBV, there is a group of rare diseases that develop in the apparent absence of immunodeficiency and are characterized by ectopic infection of EBV in T or NK lymphocytes. They are EBV-associated hemophagocytic lymphohistiocytosis (HLH), hydroa vacciniforme (HV)-like lymphoproliferative disease, and severe mosquito bite allergy (SMBA). These diseases have a particularly high incidence in East Asia and Latin America and are collectively called EBV-associated T/NK-cell lymphoproliferative diseases (EBV-T/NK-LPDs) (5, 6). However, the EBV-T/NK-LPDs concept has not been fully established. In fact, there are several unanswered questions about EBV-T/NK-LPDs. For instance, it is not known how EBV infects T and NK cells, or how they proliferate. It is also not known why EBV-infected T/NK cells are not removed by host immune responses, or whether they are immunodeficient or malignant diseases. Moreover, the scientific community cannot yet explain why the incidence rates of EBV-T/NK-LPDs are uneven across geographic territories, or whether there are any genetic predisposing factors. Finally, the ideal timing and protocol for hematopoietic stem cell transplantation for CAEBV remain to be determined.

This review investigates both original works and secondary sources on EBV-T/NK-LPDs. It is intended to stimulate discussion on the enigmatic pathogenesis and disease concept of EBV-T/NK-LPDs, to facilitate, eventually, the development of a standard therapy.

## Definitions and Brief Description of Representative EBV Diseases

EBV preferentially infects B cells via the CD21 cell surface protein, and is associated with a variety of diseases of B-cell origin. These include infectious mononucleosis, Burkitt lymphoma, diffuse large B-cell lymphoma, and Hodgkin lymphoma (7). EBV nucleic acids are detected in 25~50% of Hodgkin lymphoma in the USA and Europe (8, 9). EBV causes immunodeficiency-associated lymphoproliferative diseases, such as post-transplant lymphoproliferative disorders and lymphomas associated with HIV infection (7). EBV can infect epithelial cells and is associated with nasopharyngeal carcinoma and gastric cancer (7). EBV is also associated with diseases of T- and NK-cell origin, such as extranodal NK/T-cell lymphoma-nasal type (ENKTL), aggressive NK-cell leukemia (ANKL), and EBV-T/NK-LPDs as already mentioned. However, the specific T or NK cell receptors that contribute to the disease state are not known.

According to the 2017 World Health Organization Classification of Tumors of Haematopoietic and Lymphoid Tissues, four diseases are classified as EBV-positive T-cell and NK-cell LPDs of childhood: systemic EBV-positive T-cell lymphoma of childhood, the systemic form of CAEBV of the T/NK-cell type, HV-like LPD, and SMBA (10). ENKTL and ANKL are also described in the text and are classified under the umbrella of EBV-positive T/NK-cell proliferation. Since EBV-associated HLH is non-neoplastic, it is not listed as an EBV-positive T-cell and NK-cell lymphoproliferative disease of childhood in this classification. It is, however, important to note that EBV-associated HLH is also characterized by EBV-positive T/NK-cell lymphoproliferation. Furthermore, EBV-associated HLH is an intractable, potentially fatal disease (11). EBV-T/NK-LPD that are described and classified in the 2017 WHO lymphoma classification are shown in Table 1.

**Table 1:** 2017 WHO lymphoma classification of EBV-associated T-cell and NK-cell proliferation.

**Disease entity**	**Association with EBV (%)**	**Infected cells**	**Age group**	**Population at high risk**
Systemic EBV^+^ T cell lymphoma of childhood	100	T	Pediatric, adolescent	East Asians
Chronic active EBV infection of T/NK type	100	T, NK	Pediatric, adolescent	East Asians
Hydroa vacciniforme-like lymphoproliferative disorder	100	γδT, NK	Pediatric, adolescent	Asians, native Americans
Severe mosquito bite allergy	100	NK (T)	Pediatric, adolescent	East Asians
Aggressive NK cell leukemia	>90	NK	Adult	Asians
Extra nodal NK/T cell lymphoma, nasal type	100	NK, T	Adult	East Asians

### CAEBV of the T/NK-Cell Type, Systemic Form

CAEBV is defined as an EBV-related illness characterized by symptoms such as fever, persistent hepatitis, lymphadenopathy, hepatosplenomegaly, pancytopenia, uveitis, and interstitial pneumonia lasting for more than 3 months (3, 12). Some patients also exhibit skin-related symptoms, such as HV-like eruptions or hypersensitivity to mosquito bites. In these patients, increased amounts of EBV in the affected tissues or peripheral blood were also found. The incidence of the T/NK cell type of this disease varies markedly by race, with most cases occurring in East Asians. Most of the patients are children or young adults who exhibit clonality of EBV-infected cells. CAEBV is a potentially life-threatening illness, but the prognosis of CAEBV is variable. Some patients rapidly develop severe complications, such as multi-organ dysfunction and malignant lymphomas, whereas others remain stable without therapeutic intervention (12). Initially, CAEBV was thought to mainly be a disease of childhood, but, recently, increasing numbers of adult cases have been identified with slightly different clinical features compared to those of pediatric cases (13, 14).

### HV-Like LPD

In the 2008 WHO lymphoma classification, HV-like LPD was termed HV-like lymphoma. However, given the broad spectrum of the disease, and the lack of reliable morphological or molecular criteria, the term HV-like LPD was proposed in the 2017 classification (10). Sub-classifications of HV-like LPD include classic HV, severe (or systemic) HV, and HV-like lymphoma (15).

HV-like LPD consists of a recurrent vesiculopapular lesion with central umbilication and crust formation, which mimics herpetic vesicles usually occurring on sun-exposed areas. HV-like eruptions result from infiltration of T cells into the superficial dermis and subcutaneous tissue. In classic HV, gamma-delta T cells comprise the majority of the T cell population in the skin and mucosal epithelium (16, 17). Classic HV exhibits only skin involvement and has favorable outcomes (12, 15). The prognosis of HV-like LPD varies. Classic HV eventually resolves in adulthood. However, other types of HV-like LPD develop into a progressive disease, with worsening cutaneous symptoms and systemic dissemination (10). Patients who have systemic symptoms like fever, wastage, lymphadenopathy, and hepatosplenomegaly are categorized as severe or systemic HV.

### SMBA

SMBA is an EBV-positive NK-cell lymphoproliferative disorder defined by a hypersensitivity to mosquito bites. The disease is characterized by a high fever after mosquito bites and has various skin manifestations, including ulcers, necrosis, and scarring (18). Similar to HV-like LPD, some patients may have systemic symptoms. Most SMBA cases have been reported in Japan, with other cases reported in Korea, Taiwan, and China. Patients with SMBA have a long clinical course, with an increased risk of developing hemophagocytic syndrome and ANKL. Half of the SMBA patients were reported to have died of hemophagocytic syndrome or leukemia/lymphomas (19).

### ENKTL

ENKTL, also called nasal NK/T-cell lymphoma, is a predominantly extranodal lymphoma of NK-cell or T-cell lineage characterized by necrosis, vascular damage and destruction, cytotoxic phenotypes, and an association with EBV (20). ENKTL is more prevalent in East Asians and Native Americans in Mexico and Central/South America.

In ENKTL, the upper aerodigestive tract, including the nasal cavity and paranasal sinuses, is most commonly involved. It often progresses, exhibiting extensive midfacial destructive lesions that sometimes spread to other sites, including the skin and gastrointestinal tract. ENKTL is highly aggressive and poorly responds to therapy, resulting in low survival rates.

### ANKL

ANKL is a rare disease, but is considerably more prevalent among Asians. It involves systemic neoplastic proliferation of NK cells, frequently associated with EBV infection, and has an aggressive course (21). Patients with ANKL usually present with fever, constitutional symptoms, and a leukemic blood picture. Most cases have a fulminant clinical course, frequently complicated by multiple organ failure, coagulopathy, and hemophagocytic syndrome. The prognosis of this EBV-T/NK-LPDs is dismal.

## Etiology

The pathogenesis of EBV-T/NK-LPDs is unclear. Some evidence indicates that EBV infects T/NK cells during the primary infection (22), although this does not necessarily lead to EBV-T/NK-LPDs. EBV-infected T or NK cells may proliferate and evade apoptosis with the help of viral oncogenes and may eventually cause EBV-T/NK-LPDs in rare cases (23–25). There are however several unknown mechanisms underlying this process, as summarized in Table 2.

**Table 2:** Critical questions about the pathogenesis of EBV-associated T/NK-cell lymphoproliferative diseases.

**Question**	**Possible answer or supportive evidence**	**References**
What are the EBV receptors on T/NK cells?	1. CD21 expressed on lymphocyte progenitors 2. Transfer of CD21 from B cells to T/NK cells via immunological synapse 3. Non-CD21 receptor	1. (26, 27) 2. (28) 3. (29)
How does EBV induce T/NK-cell proliferation?	1. Involvement of the co-activating receptor CD137 2. Involvement of the cellular transcription factor NF-κB 3. Involvement of the cellular transcription factor STAT3	1. (23) 2. (24) 3. (25)
Why are EBV-infected T/NK cells not removed by cytotoxic T cells?	1. EBV-infected T/NK cells exhibit latency 2 type viral gene expression and do not express immunodominant proteins such as EBNA2 and EBNA3s	1. (3)
What explains the biased geographical distribution of EBV-T/NK-LPDs?	1. Genetic predisposition 2. Local environmental factors 3. Specific EBV strains	1. (30) 2. (31) 3. (32)
Are EBV-T/NK-LPDs immunodeficient?	1. Deficiency of EBV-specific CTLs was reported in CAEBV 2. Clinical manifestations resembling CAEBV have been documented in a patient with common variable immunodeficiency	1. (33) 2. (34)
Are EBV-T/NK-LPDs malignant?	1. Driver gene mutations identified in CAEBV	1. (2)

First, the mechanism of T/NK cell infection by EBV remains to be determined. T/NK cells lack CD21 and HLA-DR, both of which are EBV receptors in B cells. Therefore, the exact mechanism of EBV attachment and entry into T/NK cells remains largely unknown (29). Tabiasco et al. reported that NK cells activated by EBV-infected B cells acquire CD21 molecules by synaptic transfer, and these ectopic receptors allow binding of EBV to NK cells (28). EBV may attach to T cells via CD21, which is expressed in immature T cells and common progenitor cells (26, 27, 35). Recently, dual infections of both T-cell and NK-cell lineages with a single clone of EBV have been increasingly reported in patients with CAEBV. EBV may infect lymphoid progenitor cells that express CD21 and have the capacity to differentiate into both T and NK cells, at least in some patients (22).

Second, it is not known whether EBV-T/NK-LPDs are malignant neoplastic diseases like overt leukemia or lymphoma. Based on current knowledge, ENKTL and ANKL appear to be malignant neoplastic diseases. EBV-T/NK-LPDs can be considered as a pre-leukemic or lymphomatous stage in which genetic mutations accumulate and drive the process of lymphomagenesis. Recent comprehensive genetic analyses indicate that DDX3X, TP53, STAT3, and BCOR1 are driver genes that are frequently mutated in patients with ENKTL (36, 37). Interestingly, somatic driver mutations that are seen in ENKTL were found in EBV-infected cells from CAEBV patients. This suggests that EBV-infected T/NK cells evolutionally expand with driver gene mutations, and that mutations in DDX3X are important for the development of overt lymphoma and leukemia in patients with CAEBV (2). Activation-induced cytidine deaminase (AID), which belongs to the APOBEC3 protein family and induces somatic hypermutations and class switch recombination, is a candidate cause of such host gene mutations. Upregulation of AID driven by EBV infection plays an important role in c-myc translocation and tumorigenesis of Burkitt lymphoma and other lymphomas of B-cell origin (38). High expression of AID is seen in EBV-infected T/NK cells from patients with EBV-T/NK-LPDs, and may be associated with driver gene mutations (39).

Finally, although EBV is ubiquitous, it is unknown why only some people develop EBV-T/NK-LPDs in specific areas of the world. While the existence of immunodeficiencies in certain areas has been postulated, mutations in known immune-associated genes are not seen in the majority of cases (33, 34). An association with a specific HLA (A26) was reported, but it did not account for all cases (22, 30). Other explanations suggest that environmental factors are associated with the progression or development of T/NK-cell tumors. In a case-control study, exposure to pesticides and chemical solvents was correlated with ENTKL development (31). Specific EBV strains or variants that have a greater tendency to develop T/NK-cell tumors may exist (32), but there is no direct proof. Thus, the etiologies of EBV-T/NK-LPDs may be multifactorial and they are likely to consist of heterogeneous entities.

## Treatment

The prognosis of EBV-T/NK-LPDs is highly variable. Some patients rapidly develop severe complications, whereas others remain stable without therapeutic interventions. So far, no standard treatment for EBV-associated T/NK cell lymphoma has been established. Additionally, there are no effective antivirals against EBV and no molecular-targeted therapies, unlike rituximab against B cell neoplasms. The outcomes of EBV+ diffuse large B-cell lymphoma or post-transplant lymphoproliferative disorders have improved with the addition of rituximab to conventional chemotherapies (40, 41). EBV-infected T/NK cells are also generally resistant to chemotherapy, due to the expression of the multidrug resistance protein p-glycoprotein (42). To combat difficulties in treatment, hematopoietic stem cell transplantation has been introduced as a curative therapy. Although recent efforts at introducing reduced-intensity conditioning resulted in excellent transplantation outcome (43), the rates of complications associated with transplantation are high (12). It is therefore necessary to develop novel approaches to treat EBV-associated T/NK cell lymphoma.

Proteasome inhibitors, histone deacetylase (HDAC) inhibitors, and Janus kinase (JAK) inhibitors have been tested using *in vitro* or *in vivo* xenograft models as possible therapeutic approaches (25, 44–46). Additionally. immune checkpoint inhibitor therapy may be beneficial for patients with EBV-T/NK-LPDs, since programmed death-1 (PD-1) blockade with pembrolizumab has proven effective in relapsed or refractory ENKTL (47). To establish a standard therapy against EBV-T/NK-LPDs, new strategies and extensive further research are needed.

## Author Contributions

HK and SF contributed to concept development and the writing and review of this manuscript, and gave final approval of the version to be published.

### Conflict of Interest Statement

The authors declare that the research was conducted in the absence of any commercial or financial relationships that could be construed as a potential conflict of interest.
